# ALKBH3, a human AlkB homologue, contributes to cell survival in human non-small-cell lung cancer

**DOI:** 10.1038/sj.bjc.6606012

**Published:** 2011-02-01

**Authors:** M Tasaki, K Shimada, H Kimura, K Tsujikawa, N Konishi

**Affiliations:** 1 Second Department of Internal Medicine, Nara Medical University School of Medicine, Shijo-cho, Kashihara, Nara 634-8521, Japan; 2 Department of Pathology, Nara Medical University School of Medicine, Shijo-cho, Kashihara, Nara, Japan; 3 Department of Immunology, Osaka University, Graduate School and School of Pharmaceutical Sciences, Yamadaoka, Suita, Osaka, Japan

**Keywords:** lung cancer, adenocarcinoma, ALKBH3, senescence

## Abstract

**Background:**

We have demonstrated for the first time that a novel human AlkB homologue, ALKBH3, contributes to prostate cancer development, but its clinical and biological roles in lung cancer remain unclear.

**Methods:**

Expression of both mRNA and protein of PCA-1 was examined by RT–PCR and western blotting. We also assessed association with senescence and *in vivo* ALKBH3 treatment on orthotopic tumour cell inoculation, and analysed it clinicopathologically.

**Results:**

We have since found novel biological roles for ALKBH3 in human lung cancers, particularly in adenocarcinoma. Our immunohistochemical analysis of human adenocarcinomas and squamous cell carcinomas of the lung not only showed overexpression of ALKBH3 in these tumours but the percentage of cells positive for ALKBH3 also correlated statistically to recurrence-free survival in adenocarcinoma. Knockdown of ALKBH3 by siRNA transfection induced expression of p21^WAF1/Cip1^ and p27^Kip1^ in the human lung adenocarcinoma cell line A549, resulting in cell cycle arrest, senescence and strong suppression of cell growth *in vitro*. *In vivo*, peritoneal tumour growth and dissemination was inhibited in nude mice, previously inoculated with the A549 cell line, by intraperitoneal injection of ALKBH3 siRNA + atelocollagen, as demonstrated by the reduction in both number and diameter of tumours developing in the peritoneum.

**Conclusion:**

We suggest that ALKBH3 contributes significantly to cancer cell survival and may be a therapeutic target for human adenocarcinoma of the lung.

A number of investigators are engaged in examinations of the clinical, pathological and biological characteristics of both small-cell and non-small-cell lung cancers (NSCLC); yet more than 60 000 people die of lung cancer every year and the 5-year survival rate for patients with the disease remains about only 15% (Alberg *et al*, 2007; Toyoda *et al*, 2008). Adenocarcinoma accounts for approximately 70% of NSCLC, and the recent increases in the number of cases indicate an urgent need to develop not only better treatment but also improve methods of early diagnosis. The epidermal growth factor receptor (EGFR) has recently been scrutinised as a potential target in lung adenocarcinoma therapy because of its overexpression in and association with poor prognosis of many solid tumours (Meert *et al*, 2002; Hirsch *et al*, 2003). The development of EGFR inhibitors gefitinib and erlotinib initially showed dramatic effects in the treatment of lung adenocarcinoma; however, tumours frequently acquire resistance to the drugs, resulting in treatment failure (Sharma *et al*, 2007; Linardou *et al*, 2009).

DNA alkylation damage repair mechanisms are known to be controlled by six genes (*tag*, *ogt*, *ada*, *alkA*, *aidB* and *alkB*). Among them, *ada*, *alkA*, *aidB* and *alkB* are induced on exposure to a sublethal dose of alkylating agents, called the adaptive response (Sedgwick and Lindahl, 2002). In *Escherichia coli* , the *alkB* gene product was identified as protein to carry out DNA repair by oxidative demethylation (Kataoka *et al*, 1983; Dinglay *et al*, 2000; Falnes *et al*, 2002; Trewick *et al*, 2002; Aas *et al*, 2003; Sedgwick *et al*, 2007), and repairs both DNA and RNA methylation (Falnes and Rognes, 2003; Falnes *et al*, 2007). Eight AlkB homologues (ALKBH), designated hABH1 to hABH8, have since been identified in human tissues (Tsujikawa *et al*, 2007). In previous studies on prostate cancer conducted in our lab, we isolated a highly expressed protein that we originally designated as prostate cancer antigen-1 (PCA-1) (Konishi *et al*, 2005). We further characterised this protein in terms of its effects on prostate cancer cell survival and invasion through modulation of the discoidin domain receptor 1 (DDR1) (Di Marco *et al*, 1993; Vogel, 1999; Curat and Vogel, 2002; Ongusaha *et al*, 2003; Shimada *et al*, 2008). AlkB homologue-3 thus seems to participate in a wide range of biological functions involving survival and invasion of cancer cells.

In this study, we found not only overexpression of ALKBH3 in lung adenocarcinoma cells but also a correlation between expression profile and recurrence-free survival (RFS). In addition, *ALKBH3* silencing through siRNA transfection effectively induced cellular senescence and growth suppression of lung adenocarcinoma cells both *in vivo* and *in vitro.* AlkB homologue-3 may thus join EGFR as both a new molecular target in cancer therapeutics and as another useful clinicopathological marker in the management of human non-small-cell lung cancer.

## Materials and methods

### Cell culture

The non-small-cell lung cancer cell line A549, originating from a human lung adenocarcinoma, and RERF-LC-AI, originating from a human squamous cell carcinoma, were purchased from RIKEN Bio Resource Center (Tsukuba, Ibaraki, Japan) and cultured in RPMI supplemented with 10% fetal bovine serum.

### Antibodies and preparation of antisera

Antibodies to caspase-3 were supplied by PharMingen (San Diego, CA, USA), those to caspase-8 by Medical and Biological Laboratories Co., Ltd. (Nagoya, Japan), to caspase-9 by Cell Signaling Technology (Cambridge, MA, USA) and to actin by Santa Cruz Biotechnology, Inc. (Santa Cruz, CA, USA). Anti-ALKBH3 antisera were raised in rabbits against the synthetic peptide of ALKBH3 (peptide sequence NKQKSKYLRGNRNS) as an antigen. Aliquots of 0.5 mg peptide were emulsified in equal volumes of Freund’s Complete Adjuvant and injected s.c. at several sites into each rabbit. Antiserum was prepared and the relative reactivity of the antisera evaluated against the synthetic peptide by ELISA; those antisera showing high titres were affinity-purified using SulfoLink (Pierce Biotech, Rockford, IL, USA).

### Preparation of cell lysates and western blotting analysis

We resolved the cell lysates from A549 in SDS polyacrylamide gels and transferred them onto polyvinylidene difluoride membranes (Millipore, Bedford, MA, USA), which were blocked in 5% skimmed milk at room temperature for 1 h. The membranes were then incubated with each of the antibodies described in the previous section for 1 h, followed by incubation with horseradish peroxidase-conjugated anti-mouse or anti-rabbit IgG (Amersham Pharmacia Biotech, Piscataway, NJ, USA). We detected peroxidase activity on X-ray films using an enhanced chemiluminescence detection system.

### siRNA transfection of ALKBH3

Transfections were carried out using the Lipofectamine system (Invitrogen, Tokyo, Japan) in accordance with the manufacturer’s protocol. We seeded 2 × 10^6^ cells from each lung cancer cell line in 60 mm dish plates and transfected them with either 100 nmol l^−1^ of control RNA (Santa Cruz Biotechnology) or ALKBH3 siRNA. AlkB homologue-3 siRNA duplexes, generated with 3′-dTdT overhangs and prepared by Qiagen (Tokyo, Japan), were chosen against the following DNA target sequences for ALKBH3: 5′-TACCACTGCTAAGAGCCATCTCC-3′ and 5′-ACCTGCTGAGGTTCTTTGAACAC-3′.

### Tissue samples and immunohistochemistry

We obtained 86 specimens of human lung adenocarcinoma and 46 specimens of lung squamous cell carcinoma from patients at Nara Medical University Hospital. All patients provided informed consent before collection of specimens. Some patients received post-operative chemotherapy; however, no alkylating reagents such as cyclophosphamide, ifosfamide, melphalan and busulfan were administered.

The sections were incubated with the primary antibodies to ALKBH3 at 1 : 100 dilution for 16 h at 4°C and the reactions were visualised using a Histofine kit (Nichirei, Tokyo, Japan) with diaminobenzidine as the chromogen, followed by haematoxylin counterstaining. The intensity of immunohistochemical staining was evaluated at 100 × magnification (Table 1). No chemo- or radiation treatments had been performed before resection. The sections were fixed and paraffin embedded first. We investigated lung adenocarcinoma and squamous cell carcinoma diagnosed with certainty at Nara Medical University Hospital.

### Cell cycle analysis

We performed cell cycle analyses by flow cytometry as previously described (Shimada *et al*, 2003), and compared a change in the cell count in each period. All experiments were conducted at least thrice in duplicate.

### *In vivo* ALKBH3 treatment on orthotopic tumour cell inoculation

A549 (2 × 10^6^) or RERF-LC-AI cells suspended in 100 *μ*l medium were instilled into the intraperitoneal cavity of 5-week-old male BALB/c nude mice. All mice were purchased from Charles River Japan, Inc. (Kanagawa, Japan). At 7 and 14 days after the cell inoculation, we injected either control RNA or 10 *μ*mol l^−1^ of the ALKBH3 siRNA+atelocollagen (Atelogene, Koken Co., Ltd, Tokyo, Japan) mixture into groups of 11 mice. All mice were killed 28 days after injection into the intraperitoneal cavity. Tumour response was evaluated by measurement of maximum tumour diameter and number of tumours formed in the peritoneum and liver.

### Reverse transcription–PCR

Using the OneStep RT–PCR kit (Qiagen), we extracted total RNA from the homogenised A549 cell line using Trizol reagent and subjected it to reverse transcription–PCR (RT–PCR). PCR conditions were 95°C for 30 s, 55–60°C for 30 s and 72°C for 1 min through a total of 30 cycles. The PCR primer sequences for ALKBH3 were 5′-AGATGTACTGGTTCCCTGGC-3′ (sense) and 5′-CCTCACGGAACACATGGTAG-3′ (antisense). For glyceraldehyde-3-phosphate dehydrogenase (GAPDH), the primers used were 5′-ACCACAGTCCATGCCATCAC-3′ (sense) and 5′-TCCACCACCCTGTTGCTGTA-3′ (antisense). The PCR products were analysed on 1.5% agarose gel and visualised by ethidium bromide staining.

### Statistical analysis

Data were statistically analysed using the Student *t-*test or, for non-parametric analysis, the Kruskal–Wallis test. Survival analyses for biochemical recurrence were evaluated using the Kaplan–Meier method and the log-rank test. Results were considered significant at *P*<0.05.

## Results

### Expression of ALKBH3 in human NSCLC

**Figure 1:**
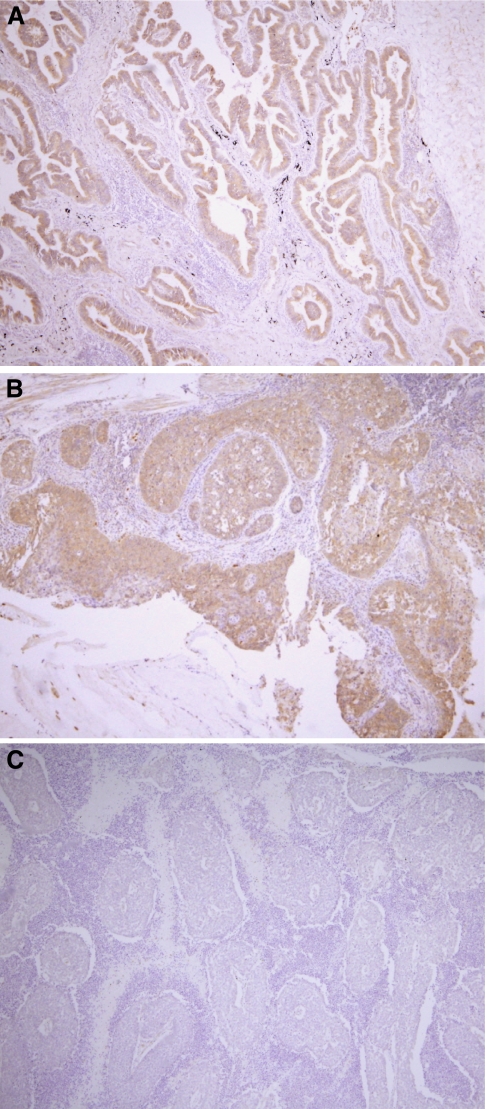
Immunohistochemical detection of ALKBH3 in lung adenocarcinoma, squamous cell carcinoma and small-cell carcinoma. (**A**) sample of adenocarcinoma. (**B**) sample of squamous cell carcinoma. (**C**) sample of small-cell carcinoma. Immunohistochemical results showed that ALKBH3 was highly expressed in adenocarcinoma and squamous cell carcinoma, but less expressed in small-cell carcinoma.

**Figure 2:**
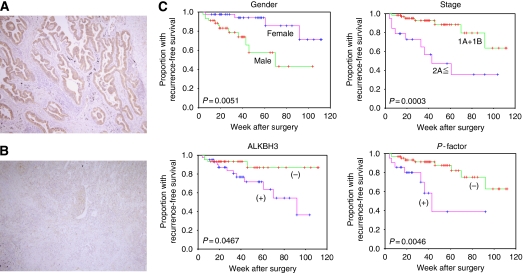
Immunohistochemical detection of ALKBH3 and Kaplan–Meier plots in adenocarcinomas. ALKBH3 is localised mainly in the cytoplasm of cancer cells. (−), <30% of immunopositive cells **A**; (+), ≥30% of immunopositive cells **B**. (**C**) Kaplan–Meier plots of recurrence-free survival in patients with lung adenocarcinoma. There was significant difference between the two groups in terms of gender, pathological stage, ALKBH3 and pleural invasion factor (*P*-factor).

Before we began the larger study, we initially performed a limited survey of the expression profile of ALKBH3 using four samples each of small-cell lung cancer, adenocarcinoma and squamous cell carcinoma (Figure 1). Immunohistochemical results showed that ALKBH3 was highly expressed in 75% of both adenocarcinoma and squamous cell carcinoma samples, but expressed to a lesser degree in only 25% of small-cell carcinomas. On the basis of these initial results, we examined the relationship between ALKBH3 expression and selected clinicopathological parameters in 132 surgical specimens of NSCLC, comprised of 86 human lung adenocarcinomas and 46 squamous cell carcinomas, in more detail (Table 1). Of those specimens, 50% of adenocarcinomas and 56.5% of squamous cell tumours demonstrated ⩾30% of cells immunopositive for ALKBH3 (Figures 2A and B). In lung adenocarcinoma only, ALKBH3 positivity was also statistically associated with recurrence-free survival and with factors such as gender, tumour stage and degree of pleural invasion (*P*-factor) (Figure 2C); these associations did not hold for squamous cell carcinoma (data not shown).

### ALKBH gene silencing and cell survival

At the beginning of the experiment, we evaluated RNA expression of ALKBH3 by real-time RT–PCR analysis as demonstrated in the following figures, but the results were not completely consistent with immunohistochemical data. AlkB homologue-3 protein in human lung-cancer cells may be stabilised by a posttranscriptional and/or post-translational mechanism including ubiquitin–proteasome signals. To confirm, we checked whether ALKBH3 was downregulated by siRNA transfection by both RT–PCR and western blotting in this study.

**Figure 3:**
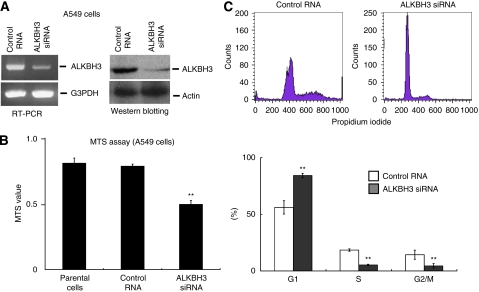
Downregulation of ALKBH3 induced cell cycle arrest in human adenocarcinoma cells. (**A**) A549 was transfected with 100 nM of ALKBH3 siRNA or control RNA. Expression levels of both mRNA and protein of ALKBH3 were examined by RT–PCR and western blotting, respectively. (**B**) Cell proliferation was quantitatively assessed by MTS assay. Columns indicate mean±s.e. (**C**) Cell cycle analysis was performed by flow cytometry using propidium iodide, as described in Materials and Methods. Columns indicate mean±s.e. ^**^P<0.05.

In the human lung adenocarcinoma cell line A549 RT–PCR and western blotting data showed that ALKBH3 gene expression was significantly reduced by transfection with 100 nM siRNA (Qiagen) for 72 h (Figure 3A). As demonstrated in Figure 3B and C, *ALKBH3* gene silencing induced cell cycle arrest at the G1 phase, resulting in inhibition of cell growth.

### Gene silencing through siRNA transfection: senescence and apoptosis

**Figure 4:**
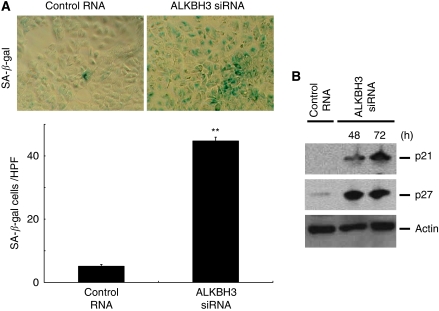
Downregulation of ALKBH3 induced senescence in human adenocarcinoma cells. (**A**) Cells were fixed and stained with SA-*β*-gal and senescence-like phenotype was visualised under a microscope ( × 400). (**B**) After 48 h or 72 h culitivation, p21 and p27 induction was examined by western blotting. They were induced in a time-dependent manner. ^**^P<0.05.

Cell cycle arrest is known to induce cytotoxicity, including cellular senescence and apoptosis. After silencing *ALKBH3* by siRNA transfection, A549 cells were found to be senescent using the cellular senescence marker, SA-*β*-gal (Figure 4A), and we detected induction of p27 and p21 in a time-dependent manner (Figure 4B); however, apoptosis was not induced as evidenced by propidium iodide staining, and cleavage in caspases 3, 8 and 9 were not observed after *ALKBH3* gene silencing (data not shown). It therefore appears that, in human lung adenocarcinoma cells, ALKBH3 knockdown inhibits cell survival, presumably through p21/p27-mediated cell cycle arrest at G1, followed by cellular senescence.

### *In vivo* effects of ALKBH3 gene silencing on tumour growth

**Figure 5:**
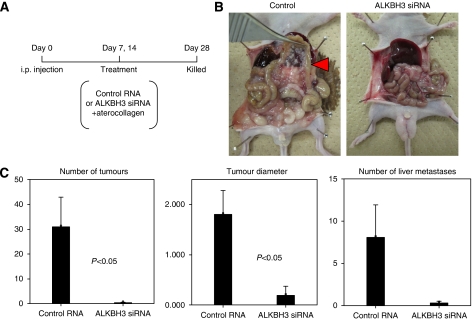
Silencing ALKBH3 reduced the peritoneal metastases of lung adenocarcinoma cells, whereas it did not reduce liver metastases. **A** and **B**, mice were given either control RNA or ALKBH3 siRNA + atelocollagen mixture at 7 and 14 days after peritoneal injection of A549 cells as described in materials and methods. The mice were killed on day 28. (**C**) Tumour size and the number of tumours in the peritoneum, as well as the number of liver metastases, were analysed. Columns, mean of 11 mice/group; bars, s.e.

To study the effects of *ALKBH3* gene silencing *in vivo,* we constructed an animal model of intraperitoneal inoculation of A549 and RERF-LC-AI cells using nude mice. At 7 and 14 days after intraperitoneal injection of cancer cells, control siRNA or ALKBH3 siRNA was intraperitoneally injected in the presence of atelocollagen as described in Materials and Methods. After 28 days, all mice were killed and tumour masses on/in the peritoneum and liver were removed and measured (Figures 5A and B). As shown in Figure 5C, the numbers of tumours formed in the peritoneum in our nude mouse model were significantly decreased in mice receiving ALKBH3 siRNA compared with mice receiving control siRNA. To validate our findings, we examined whether *ALKBH3* gene silencing *in vivo* affects squamous cell carcinoma using the human lung squamous cell carcinoma cell line RERF-LC-AI, but no significant differences in tumour formation were observed between the two groups injected with either ALKBH3 siRNA or control RNA (data not shown). The results were thus in-line with clinicopathological data, both in adenocarcinoma and squamous cell carcinoma.

## Discussion

We originally detected ALKBH3 expression in the prostate, and were able to demonstrate that a number of molecules associated with ALKBH3 were involved in cancer metastasis or resistance to anticancer drugs. In this study, we show that ALKBH3 has important roles in the survival and progression of human NSCLC cells, both *in vitro* and *in vivo.* We also tried to clarify whether ALKBH3 influences cell cycle progression and survival in human lung carcinoma.

Eight mammalian AlkB homologues (ALKBH1-8) are currently identified (Kurowski *et al*, 2003). Furthermore, in 2007, the FTO (fat mass and obesity associated) gene was found to encode a functional homologue of AlkB (Gerken *et al*, 2007; Sanchez-Pulido and Andrade-Navarro, 2007), and two more genes, TET1 and TET2 (Tahiliani *et al*, 2009; Ito *et al*, 2010), suggested possibility as a similar mechanism.

ALKBH2 and ALKBH3 share the ability of *E.coli* AlkB to directly reverse nucleic acid damage *in vitro* (Falnes *et al*, 2002; Trewick *et al*, 2002), and we reported in a recent study that ALKBH8 has important roles in the survival and progression of human urothelial carcinoma both *in vitro* and *in vivo* (Shimada *et al*, 2009). The *AlkB* family of genes is one of several that control repair of the cytotoxic damage generated in both ssDNA and RNA by S_N_2-alkylating agents; the *in vivo* function of ALKBH3 is still unclear, but it has also been shown to repair DNA and RNA basepair lesions (Dinglay *et al*, 2000; Aas *et al*, 2003). Alkylation of DNA, RNA and proteins results in induction of cytotoxic and mutagenic DNA damage, most of which is subject to excision and postreplication repair. It is well known that, in response to DNA damage, activation of either p16/Rb, p19/p53/p21 or PTEN/p27 can initiate or enhance cellular senescence (Chu *et al*, 2008), resulting in growth reduction and inhibition. DNA damage elicited in response to extracellular stresses, including exposure to chemotherapeutic drugs, can exhibit significant antitumour effects by inducing senescence, often termed premature senescence (Robles and Adami, 1998; Schmitt *et al*, 2002; Ricci and Zong, 2006); current chemotherapeutic drugs such as irinotecan, daunorubicin, hydroxyurea, retinoic acid and the previously described gefitinib are closely associated with cellular senescence through their cytotoxic effects (te Poele *et al*, 2002; Hotta *et al*, 2007). Cyclin-dependent kinase inhibitors and certain genes such as *p16*^*INK4a*^, *p21*^*WAF1/CIP1*^, *p27*^*KIP1*^ and *p53* have important roles in induction or maintenance of senescence by inhibiting the cell cycle progression at G1 arrest (Sherr and Roberts, 1999; Adams, 2009). Cisplatin induces cell cycle arrest through the p16/p53-dependent pathway in combination with increased expression of the p53 downstream effector p21, and induces characteristics of senescence rather than apoptosis. We examined whether cell death induced by ALKBH3 silencing could also be due to apoptosis, but cleavages of PARP and caspases 3, 8 and 9 were not observed, leaving us to infer that senescence was the underlying cause of growth inhibition and death. Senescence has been shown to be involved in antitumour effect by various anticancer agents and by ionising radiation (Wainwright *et al*, 2001; Han *et al*, 2002; Mansilla *et al*, 2003). In this study, we evaluated the role of ALKBH3 in cancer cell survival, but not in the sensitivity to anticancer drugs including alkylating reagents. Knockdown experiments using siRNA revealed that ALKBH3 contributes to lung adenocarcinoma cell growth through accelerating G1/S transition. As generally accepted, cancer cells arrested at G1 phase are much more sensitive to DNA damaging reagents; therefore, ALKBH3 may be one of the key molecules that determine chemotherapeutic efficacy in lung adenocarcinomas. We further examine whether *ALKBH3* gene overexpression or downregulation affects chemo- or radiosensitivity. However, the surgical specimens were not exposed to chemotherapeutic drugs.

We found that silencing ALKBH3 through siRNA transfection significantly inhibited cell growth in human NSCLC *in vitro* and *in vivo* and that, in culture, p21 and p27 were upregulated following *ALKBH3* knockdown. We know that p21 binds to CDK2, inhibiting kinase activity in various types of cancer cells and inducing cell cycle arrest at G1 with subsequent cellular senescence (Sherr and Roberts, 1995, 1999; Chang *et al*, 2000). p21 protein or mRNA is regulated at both the transcriptional and posttranscriptional levels. Among the transcription factors that increase p21 mRNA levels are Sp1, Sp3, E2Fs, STATs and AP2; in addition, p21 transcription is upregulated in response to DNA damage and to p53-mediated tumour suppressor signals (Gartel and Tyner, 1999). p27, on the other hand, inhibits the catalytic activity of CDK4, also resulting in cell cycle arrest at G1 – and eventual senescence – through phosphorylation of Rb protein (Sherr and Roberts, 1995; Alexander and Hinds, 2001). However, we did not find significant modulation in A549 cells of either p53 or Rb protein in response to *ALKBH3* gene silencing (data not shown). Of course, we acknowledge the possibility that any DNA damage occurring after *ALKBH3* silencing in lung adenocarcinoma cells may induce transcriptional factors other than p53/Rb that upregulate both p21 and p27, and we cannot deny the possibility that the ubiquitin–proteasome protein degradation pathway may be partly involved in p21, p27 induction.

Immunohistochemistry clearly showed that ALKBH3 was highly expressed in human NSCLC, both in adenocarcinomas and in squamous cell carcinomas, but ALKBH3 expression profiles correlated, in a statistically significant manner, to recurrence-free survival only in cases of adenocarcinoma, implicating ALKBH3 expression as a useful diagnostic and prognostic factor for adenocarcinoma outcome. At present, it is unclear to us as to why ALKBH3 would be more highly expressed in non-small-cell cancers than in small-cell lung carcinomas, although, in studies by other investigators, high ALKBH3 expression has been detected in adenocarcinomas of other organs, such as prostate and colon (Tasaki M *et al*, manuscript in preparation), which, therefore, raises the possibility that expression may be associated specifically with glandular epithelial tumourigenesis. The different environmental and genetic backgrounds of tumours may also explain expression variation; squamous cell carcinoma is highly associated with smoking history and male gender, whereas adenocarcinomas frequently show *EGFR* mutations and occur more often in women. Cancer location in terms of tumour microenvironment may also have a role – adenocarcinomas tend to be located in the peripheral lung, whereas squamous cell carcinomas arise preferentially from hilar regions. The presence and percentage of ALKBH3-positive cells might offer a meaningful way to predict/detect cancer recurrence at an earlier stage; moreover, targeting therapy to the *ALKBH3* gene might markedly improve the clinical outcome for patients with adenocarcinoma. There is precedence in that mutations in both *ras* and *EGFR* in NSCLC have recently been used in the clinical setting to predict outcome; in fact, patients whose tumours show *K-ras* mutations, with or without increased *EGFR* copy number, have been shown to have a >96.5% chance of disease progression (Massarelli *et al*, 2007; Kalikaki *et al*, 2008). However, *EGFR* and *ras* mutations are not a common feature of many tumours; our data strongly suggest that *ALKBH3* would be an important marker and target in a wide range of cancers.

In summary, ALKBH3 is overexpressed in NSCLC and has an important role in carcinogenesis. *ALKBH3* gene silencing inhibits cancer cell survival, and targeted downregulation, as was carried out in our *in vivo* study by injection of a siRNA and atelocollagen cocktail, could be a novel clinical tool for lung cancer therapy. Our immunohistochemical analysis further suggests that the ALKBH3 expression profile of tumours may be a predictive factor for tumour recurrence of adenocarcinoma, in particular, and may also join *EGFR* mutational analysis as a marker for sensitivity to chemoradiation.

## Figures and Tables

**Table 1:** Clinicopathologic characteristics in lung adenocarcinoma and squamous cell carcinoma

	**Adenocarcinoma**	**Squamous cell carcinoma**
*Gender*		
Male	45	39
Female	41	7
		
*Age*		
<70	41	12
70⩽	45	34
		
	**Median 71 years range 48–91**	**Median 75 years range 56–88**
*Tumour diameter*		
<30 mm	55	24
30 mm⩽	31	22
		
*Stage*		
IA	46	22
IB	21	12
IIA	5	6
IIB	3	5
IIIA	11	1
IIIB	0	0
IV	0	0
		
*Vascular invasion*		
(+)	26	8
(−)	60	38
		
P*-factor*		
(+)	21	14
(−)	65	32
		
Total	86	46
